# Preselected and preferred immersive virtual reality versus narrative alone to induce post‐stress relaxation in patients with pulmonary arterial hypertension: A pilot study on perceived stress and heart rate

**DOI:** 10.1111/bjhp.70059

**Published:** 2026-02-09

**Authors:** Alessandra Gorini, Beatrice De Maria, Sara Checchia, Roberta Maioli, Martina Vigorè, Patrycja Krasinska, Maurizio Bussotti, Luca Bernardelli, Laura Adelaide Dalla Vecchia, Stéphane Bouchard

**Affiliations:** ^1^ Dipartimento di Scienze Cliniche e di Comunità, Dipartimento di Eccellenza 2023‐2027 Università degli Studi di Milano Milan Italy; ^2^ Istituti Clinici Scientifici Maugeri IRCCS, PsyCaRe Lab Milan Italy; ^3^ Istituti Clinici Scientifici Maugeri IRCCS Milan Italy; ^4^ PhD Program HuME ‐ The Human Mind and its Explanations: Language, Brain and Reasoning Scuola Universitaria Superiore IUSS Pavia Italy; ^5^ Become S.r.l. Milan Italy; ^6^ Département de psychoéducation et de psychologie Université du Québec en Outaouais Gatineau Canada; ^7^ Centre de recherche du Centre Intégré de Santé et de Services Sociaux de l’Outaouais Gatineau Canada

**Keywords:** heart rate, immersive virtual reality, pulmonary arterial hypertension, rehabilitation, relaxation, stress

## Abstract

**Objectives:**

Several studies have shown the effectiveness of immersive virtual reality (IVR)‐based relaxation techniques in alleviating stress within the general population. However, few data are available on patients, or on the effectiveness of different scenarios in inducing relaxation. This pilot study aims to compare the effectiveness of three relaxation techniques—preselected IVR (IVR‐PS), preferred IVR (i.e. chosen by the participant from different alternatives—IVR‐PR), and narrative alone (CTR)—in reducing physiological and psychological stress in 16 pulmonary arterial hypertension (PAH) female patients (*N* = 16, average age: 46 ± 10.66 years; average education: 13.31 ± 3.8 years; mean duration of illness: 8.56 ± 5.24 years) following an acute stress.

**Methods:**

Patients performed a mental stress test followed by three different relaxation sessions presented in a randomized order on three separate occasions. Self‐perceived stress, level of relaxation, and heart rate (HR) were monitored during the sessions. Participants' ratings of their experiences were also collected.

**Results:**

The results indicated that the three relaxation methods were equally effective in reducing perceived stress induced by acute stress and in lowering HR. However, greater cognitive activation was reported in the two IVR conditions compared with the narrative condition.

**Conclusions:**

This is the first study to show evidence of the impact of IVR on a rare population. Despite the lack of significant differences between the two IVR and narrative‐alone conditions in physiological and subjective relaxation, more than half of the participants expressed a subjective preference for the virtual experience, especially for the preferred one.

**Statement of Contribution**

**What is already known?**

- Immersive virtual reality (IVR) has been shown to reduce stress and promote relaxation in healthy populations, though evidence in clinical groups remains limited.
- Patients with pulmonary arterial hypertension (PAH) experience autonomic dysregulation and psychological distress. However, referral to, participation in, and engagement with non‐pharmacological relaxation strategies remain low in this group.
- Personalization and user‐centred design in IVR may enhance engagement and perceived effectiveness, yet this has rarely been tested in patient populations.

**What does this study add?**

- This is the first study to compare IVR‐based relaxation (both preselected and participant‐preferred scenarios) with traditional narrative relaxation in patients with PAH.
- It demonstrates that IVR relaxation is feasible, well accepted, and comparable in efficacy to traditional techniques for reducing stress and heart rate following acute stress induction.
- Findings highlight that personalization enhances user engagement and perceived quality, informing the design of future randomized controlled trials of IVR in clinical rehabilitation contexts.

## INTRODUCTION

Pulmonary arterial hypertension (PAH) is a rare, progressive disorder that occurs 3–5 times more frequently in females than in males. It is characterized by increased pulmonary vascular resistance and is often associated with autonomic imbalance, which can lead to increased disease severity (Latus et al., 2015; Tang et al., 2021; Tsai et al., 2019) and mortality (Thayer et al., 2010; Thayer & Lane, 2007). Autonomic dysregulation is also often associated with psychosocial, cognitive, and emotional impairments, such as anxiety, depression, and reduced ability to cope with stress (Chalmers et al., 2014; Kemp et al., 2010; Kemp & Quintana, 2013; Koch et al., 2019; Lischke et al., 2018). Autonomic dysregulation and a diminished capacity to manage stress can pose a considerable challenge for individuals with PAH, ultimately worsening their overall health and well‐being.

As pharmacological treatments for PAH have not been shown to improve autonomic function (Hemnes & Brittain, 2018; Vaillancourt et al., 2017), various psychological interventions such as cognitive behavioural therapy (CBT), mindfulness‐based stress reduction (MBSR), relaxation training, and structured counselling have been employed to alleviate symptoms of anxiety and depression and enhance perceived quality of life in patients with PAH (Mai et al., 2022; Rawlings et al., 2022; Tarantino et al., 2020). Relaxation techniques, in particular, have been shown to improve cardiac response (Benz et al., 2022; Nesvold et al., 2012) and individual well‐being (Niazi & Niazi, 2011; Rosenkranz et al., 2016; Spijkerman et al., 2016). However, participation and engagement in these interventions remain low. Studies report that only around 24% of PAH patients with mental health needs receive psychological or psychopharmacological treatment, and attendance at group‐based interventions like MBSR is often poor due to barriers such as lack of awareness and travel difficulties (Bussotti & Sommaruga, 2018). Furthermore, engagement may also be hindered by symptom burden (e.g., dyspnoea and fatigue), competing self‐management demands, and motivational constraints.

In recent years, traditional relaxation techniques based on relaxing narratives have often been replaced by immersive virtual reality (IVR) technology, which has been proven effective in reducing stress and increasing the ability to cope with anxiety (Bouchard et al., 2012; De Luca et al., 2019; Failla et al., 2022; Gorini et al., 2010; Ma et al., 2023; Manzoni et al., 2008; Riva et al., 2021; Spano et al., 2023; Tarrant et al., 2018). This technology mitigates some barriers to participation and engagement by providing a highly controlled and compelling sensory environment that reduces exteroceptive and interoceptive distractors (Berthiaume et al., 2021), increases attentional focus, and promotes relaxation. Mechanistically, IVR leverages presence—the subjective experience of ‘being there’—which can enhance narrative delivery by binding guided imagery to congruent visual and auditory cues while reducing off‐task mind wandering (Gorini et al., 2011; Triberti et al., 2025). Various studies have shown that experiencing IVR environments that resemble natural environments can improve relaxation and heart rate (HR) response more effectively than traditional relaxation techniques (Anderson et al., 2017; Annerstedt et al., 2013; Knaust et al., 2022; Liszio et al., 2018; Riches et al., 2023; Valtchanov et al., 2010).

To complement this, user‐centred virtual reality (VR) emphasizes aligning content with users' preferences and goals. In the context of relaxation, for example, showing preference‐congruent natural scenes may strengthen acceptance, perceived relevance, reuse intentions, and long‐lasting effects, allowing participants to focus on the visual and auditory anchors of their choice, while reducing the scope of the content to which users' minds wander (Pardini et al., 2022; Pizzoli et al., 2019; Seabrook et al., 2020; Vaquero‐Blasco et al., 2021). This could be particularly useful for users who have difficulties focusing their attention, lack the motivation to practice mindfulness or other relaxing techniques, and/or have no prior experiences with such practices.

Such data suggest that user‐centred, IVR‐based relaxation techniques could be used as a non‐pharmacological approach to increase patients' ability to relax and decrease their physiological activation in response to stressful events. Based on these findings, we conducted a pilot study to compare the effectiveness of three relaxation techniques—preselected IVR (IVR‐PS), preferred IVR (i.e., chosen by each participant among different alternatives—IVR‐PR), and narrative alone (CTR)— in reducing physiological and psychological stress in female patients with PAH.

## MATERIALS AND METHODS

### Participants

Sixteen female patients with chronic PAH who were undergoing an in‐hospital rehabilitation programme participated in the study. Inclusion criteria included a diagnosis of PAH, being aged 18 or over, having normal or corrected‐to‐normal vision and hearing, having been on stable PAH therapy for at least 6 months, and understanding Italian. Patients were excluded if they had atrial fibrillation, serious mental illness, were taking psychotropic medication, had ascertained cognitive deficits, photosensitive epilepsy, vestibular and/or balance disorders, or had previously experienced severe simulator sickness.

### Measures

#### Psychological assessment

- *Short‐form Depression Anxiety and Stress Scale (DASS‐21)* (Bottesi et al., 2015). The DASS‐21 was administered at the beginning of the three experimental sessions (i.e., the CTR session, the IVR‐PS session and the IVR‐PR session) to assess the presence of depressive, anxious, and/or stressful symptoms that could interfere with relaxation.
- *Smith Relaxation State Inventory 3 (SRSI3)* (Smith, 2000). The SRSI3 was administered at the beginning of the three experimental sessions and immediately after the three relaxation phases to measure the following relaxation‐related dimensions: rest/refresh, energized, physical relaxation, at ease/peace, joy, mental quiet, awareness, somatic stress, emotional stress, and cognitive stress.
- *Subjective Units of Distress Scale (SUDS)* (Wolpe, 1969). The SUDS was administered to assess participants' self‐perceived stress levels at various points during each session (before the stress test, at the end of each block of the Montreal Imaging Stress Task (MIST), and after relaxation).

#### Questionnaires related to the IVR experience

- *Simulator Sickness Questionnaire (SSQ)* (Bouchard et al., 2021). The 16‐item SSQ was used to assess participants' levels of simulator sickness before and after the IVR interventions.
- *IVR Engagement*. A total of three general system feedback relating to the patient's perceived level of engagement, quality, and future use were provided after the two IVR sessions.
- *Visual Imagery Questionnaire (VVIQ)* (Marks, 1973). The VVIQ was used to assess patients' mental imagery capacity by prompting them to create a mental image of the people, objects, or settings presented in each scenario.

#### Physiological assessment

Patients were instrumented with a portable electrocardiogram (ECG) recording device (Faros 360°, MegaElectronics, Bittium, Kuopio, Finland) with a sampling rate fixed at 500 Hz for the entire duration of each experimental session.

The HR was calculated from the recorded ECG signal for each experimental phase considering the whole duration of each phase.

### Experimental procedure

**FIGURE 1:**
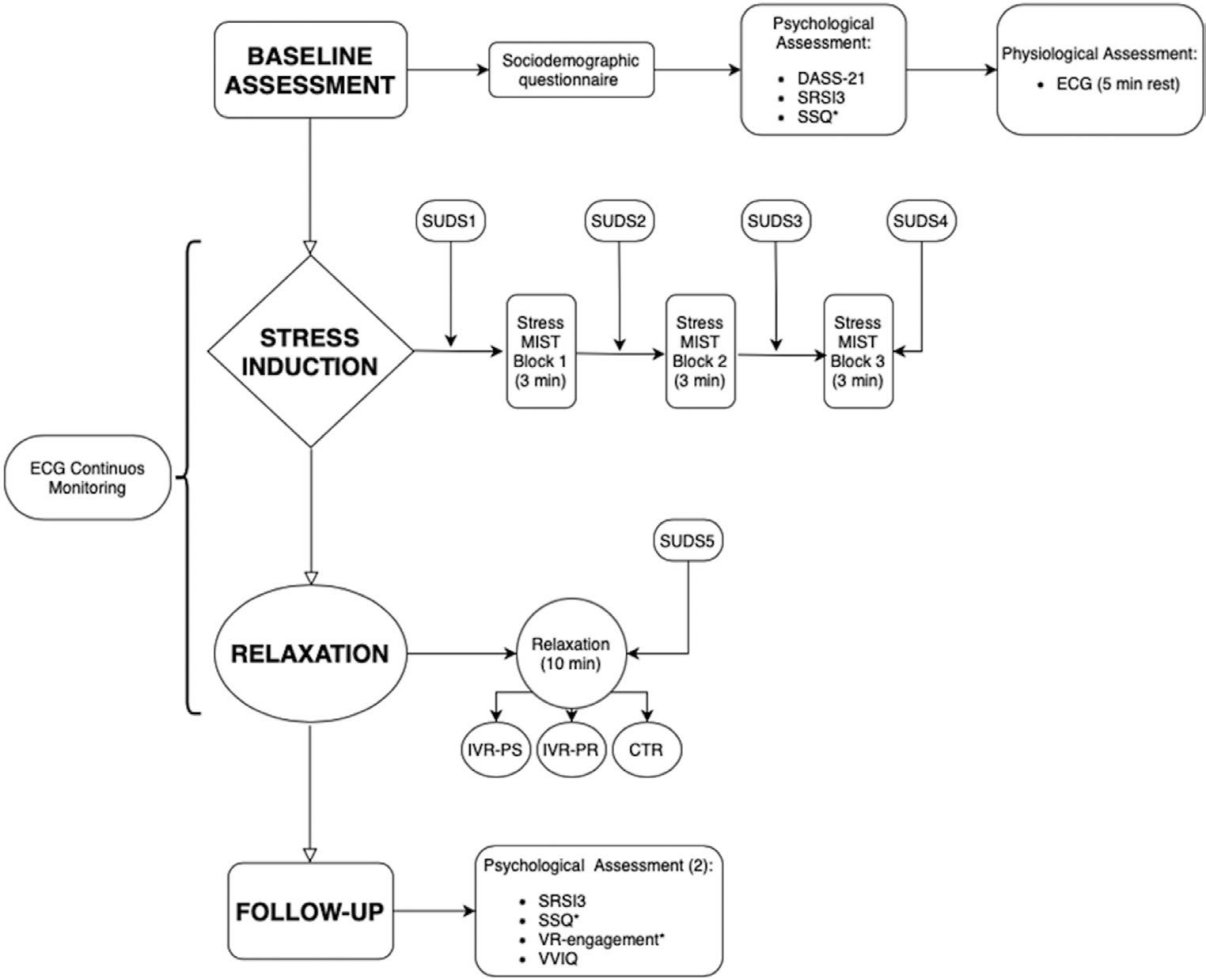
Flow chart of the study design. CTR, control condition (narrative only); DASS‐21, Depression Anxiety and Stress Scale; IVR‐PR, immersive virtual reality—preferred; IVR‐PS, immersive virtual reality—preselected; MIST, Montreal imaging stress task; SRSI3, Smith Relaxation State Inventory; SSQ, Simulator Sickness Questionnaire; SUDS, Subjective Units of Distress Scale; VVIQ, Visual Imagery Questionnaire.

The study utilized a randomized crossover design, as illustrated in Figure 1. Each patient underwent the three experimental sessions (i.e., the CTR session, the IVR‐PS session and the IVR‐PR session) in a randomized order, with a 1‐week interval between each session. Each session comprised four phases: *baseline, stress, relaxation*, *and follow‐up*.

Stress induction consisted of administering the Montreal imaging stress task (MIST) (Dedovic et al., 2005), a computerized math task that has been proven by various studies and samples to induce psychological stress (Brugnera et al., 2018; Han et al., 2017), including in patients with PAH (Gorini et al., 2023). Three blocks of arithmetic operations lasting three minutes each were presented. To increase the participants' stress, the experiment supervisor verbally applied pressure on three separate occasions during the test. To measure the perceived level of stress, the SUDS was administered at the end of each block.

After the stress induction, the *relaxation* phase began and the following three different relaxation sessions were randomly provided to each patient on three different days:

#### Relaxation based on preselected immersive virtual reality scenario (IVR‐PS)

**FIGURE 2:**
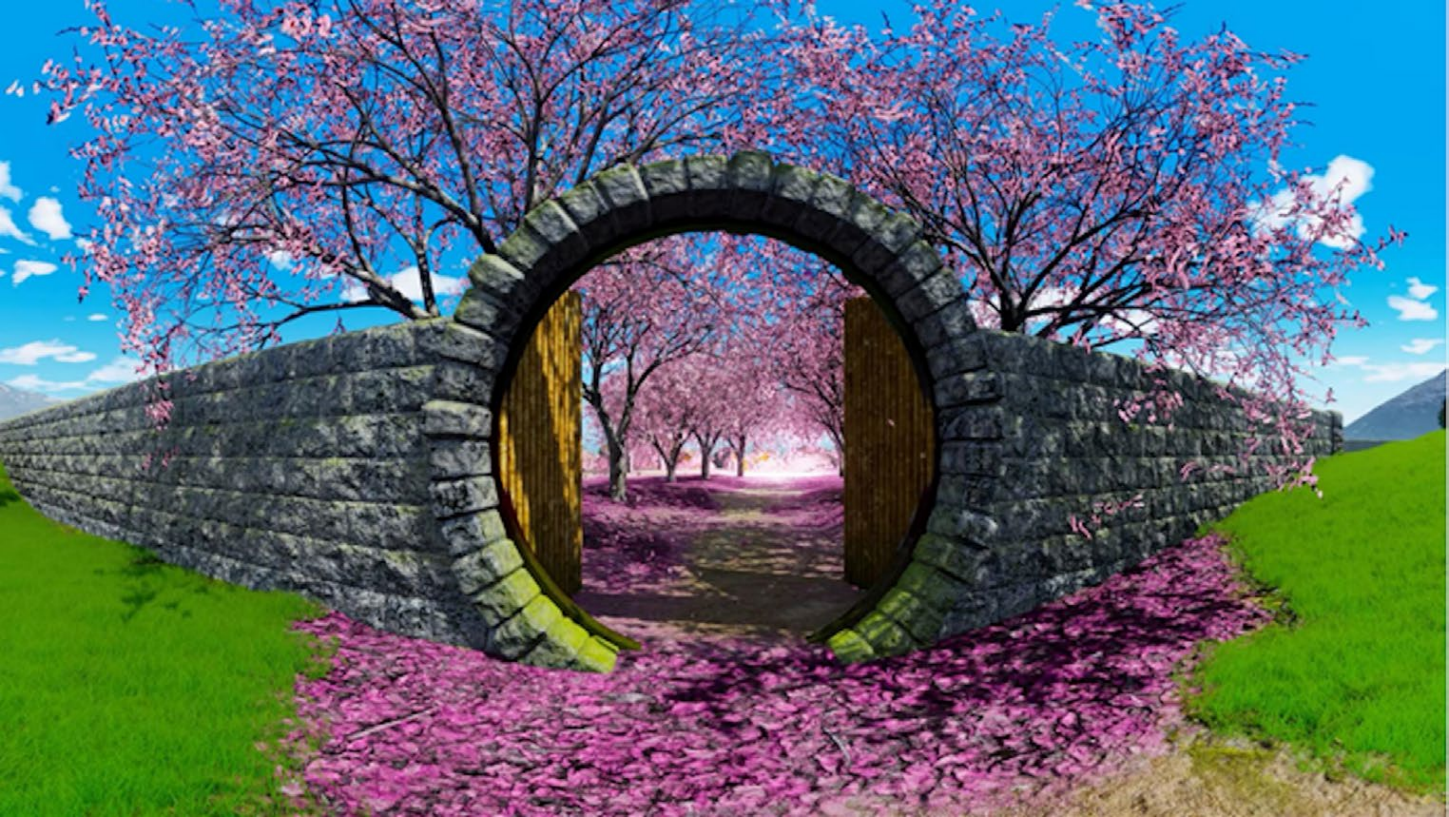
A screenshot from the ‘Secret Garden’.

This condition was based on ‘The Secret Garden’ (Figure 2), a 360 VR relaxing environment (Meyer et al., 2022; Pallavicini et al., 2022; Riva et al., 2020, 2021) developed by the Italian Company BECOME (https://www.discoverbecome.com/) (for a detailed description of the environment see (Riva et al., 2021)).

#### Relaxation based on preferred immersive virtual reality scenario (IVR‐PR)

**FIGURE 3:**
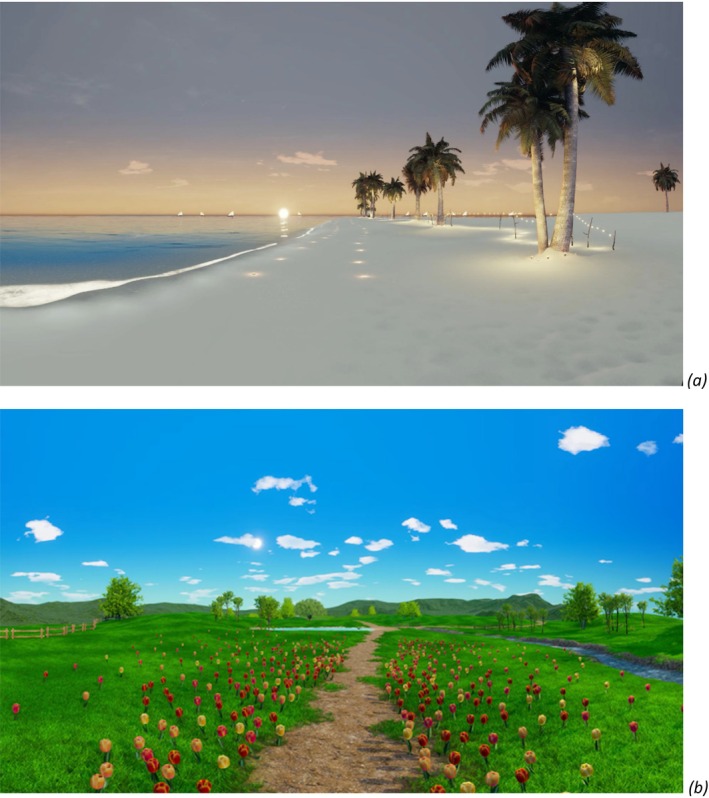
Screenshots from the IVR‐PR condition (a) ‘The Beach at Sunset’; (b) ‘The Waterfall in the Prairie’.

The IVR‐PR condition consisted of the presentation of an IVR environment chosen by each patient from two different natural‐like virtual contexts (e.g., a white beach or a countryside) (Figure 3a,b), which were developed by the same company that worked on the ‘Secret Garden’. Before the relaxation phase, participants were shown still images and brief text descriptions of the two scenes. No headset preview was provided to avoid pre‐exposure effects. Participants then selected their preferred scene. Reasons for scene choice were not systematically collected.

Each of the two virtual environments was accompanied by a relaxing narrative with a similar structure to that of the ‘Secret Garden’, but with information specific to stimuli in the chosen scene (e.g., about ocean waves).

#### Relaxation based only on a relaxing narrative (CTR)

The CTR condition involved a relaxation session with a recorded narrative by a male narrator, as in the IVR conditions, accompanied by relaxing music. Created by a psychologist, the narrative included breath control and relaxation visualizations similar to those in the IVR sessions, plus a mental imagery component. As in the IVR conditions, there were periods of unguided practice lasting up to 40 s. Three blind assessors (patients with PAH not included in the study) rated the three narratives on their relaxing effect, pleasantness, and length using a 0–10 VAS, with scores ranging from 9 to 10, indicating comparability.

Each session lasted 10 minutes and took place in a soundproof room. During the two IVR sessions, participants wore the Oculus Go headsets and could enjoy the 360‐IVR environment, rotating as desired.

The post‐assessment phase concluded the session, during which participants rated their perceived stress using the SUDS and evaluated their state of relaxation using the SRSI3 questionnaire. Additionally, the patient's mental imagery capacity was assessed using the VVIQ questionnaire. After the IVR sessions, engagement levels and simulator sickness were also examined.

#### Institutional Review Board (IRB) approval

The protocol adhered to the standards outlined in the Declaration of Helsinki and was approved by the Ethics Committee of the hospital in which the study has been conducted (approval number: 2694 CE). Participants signed an informed consent prior to participating.

### Data analysis

Descriptive statistics were used to analyse the descriptive variables characterizing the sample. Categorical variables were presented as absolute numbers (percentage), while continuous variables were presented as mean ± standard deviation. Repeated measures ANOVAs were performed to test the differences in the ECG‐derived, psychological, and performance variables within each experimental condition and between the three relaxation techniques. The analyses were performed using (Sigmaplot, Systat Software, Inc., Chicago, IL, USA, version 11.0).

For repeated measures ANOVAs, we reported partial eta squared (*ηp* ^2^) as a measure of effect size.

For paired comparisons, we reported Hedges' g (with 95% Cls).

## RESULTS

The patients' mean age was 46 ± 10.66 years; their mean level of education was 13.31 ± 3.8 years and the mean duration of illness calculated from the diagnosis was 8.56 ± 5.24 years.

### Baseline psychological measurements across conditions

No significant differences were found in depressive, anxious, and stress symptoms before the three sessions (DASS Depression: IVR‐PS = 4.75 ± 6.23; IVR‐PR = 5.13 ± 5.51; CTR = 5.63 ± 5.28; *F* (2, 30) = 0.25, *p* = 0.78, *ηp* ^2^ = 0.16. DASS Anxiety: IVR‐PS = 6.37 ± 7.34; IVR‐PR = 6.62 ± 5.04; CTR = 6.12 ± 6.79; *F* (2, 30) = 0.08, *p* = 0.92, *ηp* ^2^ = 0.06. DASS Stress: IVR‐PS = 10.38 ± 7.49; IVR‐PR = 13 ± 6.85; CTR = 10 ± 6.41; *F* (2, 30) = 1.45, *p* = 0.25, *ηp* ^2^ = 0.09. DASS Total: IVR‐PS = 10.75 ± 8.92; IVR‐PR = 12.37 ± 6.61; CTR = 10.88 ± 7.53; *F* (2, 30) = 0.48, *p* = 0.62, *ηp* ^2^ = 0.31).

### Effects of stress and relaxation within and between conditions

**FIGURE 4:**
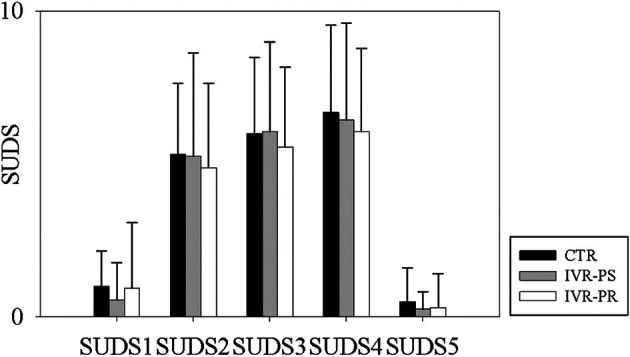
Mean scores of SUDS throughout the three conditions.

**FIGURE 5:**
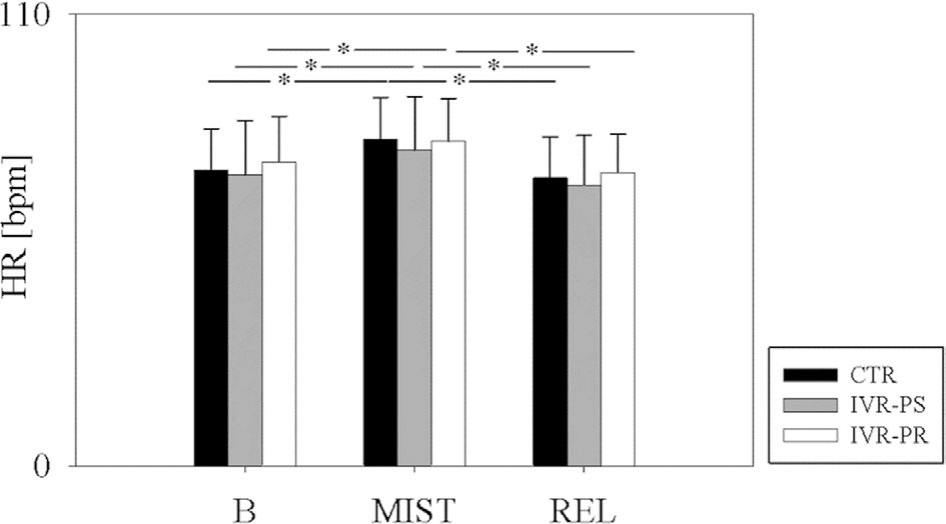
Variation of HR throughout the three conditions.

Significant changes in perceived stress levels (measured using the SUDS, *F* (4,120) = 75.89, *p* < 0.001, *ηp* ^2^ = 0.563) and HR (*F* (2, 60) = 34.68, p < 0.001, *ηp* ^2^ = 0.10) were observed in all three conditions increasing from the baseline to the MIST (with the highest peak detected at the end of the third block) and decreasing from the MIST to the end of the relaxation periods (see Figures 4 and 5).

**FIGURE 6:**
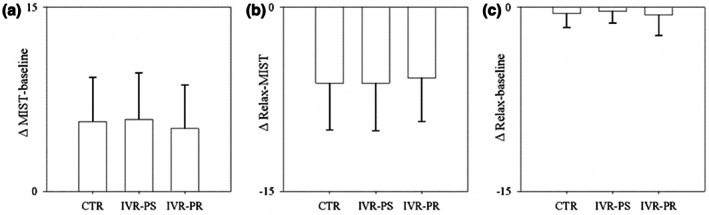
SUDS delta in the three conditions (a) MIST and baseline; (b) MIST and Relax; (c) Baseline and relax.

No significant differences were observed among the three conditions for the different SUDS deltas (to calculate the deltas we only considered SUDS1, SUDS4, and SUDS5, since SUDS4 represents the highest value after stress induction):

- MIST—Baseline (*F* (2, 30) = 0.74, *p* = 0.467, *ηp* ^2^ = 0.014) (Figure 6a);
- MIST—Relax (*F* (2, 30) = 0.44, *p* = 0.599, *ηp* ^2^ = 0.006) (Figure 6b);
- Baseline—Relax (*F* (2, 30) = 0.26, *p* = 0.707, *ηp* ^2^ = 0.011) (Figure 6c).

**FIGURE 7:**
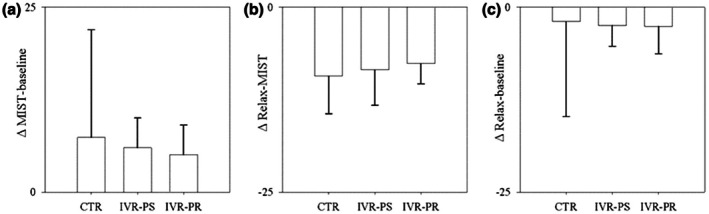
HR delta in the three conditions (a) MIST and baseline; (b) MIST and Relax; (c) Baseline and Relax in the three conditions.

Similar results have been found in the HR variation (ΔMIST‐relax *F* (2, 30) = 1.77, *p* = 0.472, *ηp* ^2^ = 0.11; ΔBaseline‐MIST *F* (2, 30) = 0.42, *p* = 0.646, *ηp* ^2^ = 0.03; ΔBaseline‐relax *F* (2, 30) = 0.74, *p* = 0.472, *ηp* ^2^ = 0.047) (Figure 7a–c).

Analysing the delta between the pre‐ and post‐SRSI3 values, we found no significant differences in any of the subscales except for the ‘cognitive stress’, which was higher in the IVR‐PS condition (mean = 2.03 ± 1.77) than in the CTR condition (mean = 0.94 ± 1.56) (*p* < 0.05) (see Table 1).

**TABLE 1:** Analysis of the SRSI3 questionnaire.

Subscale	Mean ± SD IVR‐PS	Mean ± SD IVR‐PR	Mean ± SD ctr	*F* (2, 30)	*p*‐value	*ηp* ^2^
Feeling refreshed	0.75 ± 1.39	1.44 ± 1.93	0.56 ± 1.03	2.25	0.12	0.13
Feeling energized	0.25 ± 0.77	0.88 ± 1.59	0.81 ± 1.05	1.54	0.23	0.09
Physical relaxation	2.22 ± 1.99	2.72 ± 1.81	1.53 ± 2.89	2.68	0.09	0.15
Feeling at ease	0.54 ± 0.78	0.56 ± 1.26	0.33 ± 0.77	0.31	0.73	0.02
Joy	0.31 ± 0.63	0.75 ± 0.98	0.62 ± 1.16	1.48	0.25	0.09
Mental quiet	0.81 ± 1.12	1.34 ± 1.66	1.00 ± 0.98	0.66	0.51	0.04
Awareness	0.25 ± 1.00	1.13 ± 1.82	0.38 ± 1.31	2.17	0.13	0.13
Somatic stress	0.58 ± 0.79	0.52 ± 1.39	0.44 ± 0.90	0.10	0.90	0.01
Emotional stress	0.29 ± 0.76	0.67 ± 0.97	0.21 ± 0.70	2.03	0.15	0.12
Cognitive stress	2.03 ± 1.77	1.81 ± 1.63	0.94 ± 1.56	3.16	0.06	0.17

Abbreviations: CTR, control condition (narrative only); IVR‐PR, immersive virtual reality—preferred; IVR‐PS, immersive virtual reality—preselected.

### Simulator sickness

Simulator sickness did not change from pre to post nor in the IVR‐PS condition, *t* (15) = −0.05, *p* = 0.06, *g* = −0.01, 95% CI [−0.48, 0.45] and in the IVR‐PR condition, *t* (15) = 1.85, *p* = 0.86, *g* = 0.44, 95% CI [−0.06, 0.92].

### Ratings of the IVR experiences

Most participants (12/16, 75%) rated the IVR‐PS experience as extremely engaging, while the others found it very engaging (4/16, 25%). Most of them rated the quality of the experience as extremely high (13/16, 81% versus very high 3/16, 19%) and expressed extreme willingness to use it again (12/16, 85% versus very willing 4/16, 25%).

Similar results were reported after the IVR‐PR experience that was judged as extremely engaging by 14/16 participants (88%), extremely high quality by 13/16 participants (81%), and extremely likely to be used again by 12/16 participants (75%).

At the end of the last session, participants were asked to indicate their preferences regarding the various relaxation experiences by selecting the most relaxing and enjoyable one. The majority favoured the IVR relaxation over the relaxation technique not relying on VR (10/16, 63%), while the rest did not express a preference or opted for the narrative (3/16, 19%). Furthermore, the preferred scenario was found to be more relaxing and enjoyable than the ‘Secret Garden’ by 81% (13/16).

### Vividness of mental imagery

Finally, the vividness of mental imagery was assessed. The results showed a mean score of 59.47 ± 12.07, indicating that none of the participants displayed a significant inability to visualize mental images (aphantasia). No correlations were found between the VVIQ score and the perceived stress after relaxation in the three conditions (*r* = −0.115; 0.192; 0.433; *p* = 0.671; 0.476; 0.094 for the IVR‐PS, IVR‐PR, and CTR conditions, respectively).

### Internal consistency

Internal consistency in our sample was acceptable to excellent (Cronbach's *α* = .65–.84 across DASS‐21 subscales; *α* = .89 for DASS total; *α* = .91 for SRSI‐3 total; *α* = .84 for SSQ; *α* = .92 for VVIQ).

Given the limited sample size, α values should be interpreted with caution; nonetheless, internal consistencies were comparable to those reported in prior validations.

## DISCUSSION

This study investigated the effects of two IVR environments and a narrative on relaxation in PAH inpatients undergoing a rehabilitation programme. Participants first completed a stress‐inducing task and then experienced the three relaxation conditions. The results showed a similar reduction in stress in all conditions, suggesting that IVR and narrative are equally effective in reducing both physiological and mental stress.

These findings are consistent with those of previous studies involving healthy volunteers (Gaggioli et al., 2020; King et al., 2024; Modrego‐Alarcon et al., 2021; Naylor et al., 2019), which did not find any significant differences between IVR relaxation and traditional techniques. However, our results contrast with those of three previous studies (Mistry et al., 2020; Navarro‐Haro et al., 2017; Yildirim & O'Grady, 2020), which suggested that IVR is more effective than narrative techniques alone in inducing relaxation. A possible explanation for our results is related to the presence of PAH itself. As well as frequently experiencing unpleasant and worrying symptoms such as shortness of breath, fatigue, chest pain, and dizziness, living with a chronic and potentially life‐threatening condition such as PAH can cause emotional distress, anxiety, and uncertainty about the future. Additionally, managing the necessary treatments, medications, and lifestyle adjustments can contribute to stress for patients with this rare disease. For these reasons, we hypothesize that these patients are hyperactivated both from a psychological and physiological point of view. This condition may make the benefits of different relaxation techniques particularly significant for them, rendering the traditional narrative‐based approach as effective as the more innovative and engaging methods, such as IVR.

An alternative explanation can be found in the high degree of immersion and engagement provided by IVR, which makes it inherently arousing (Felnhofer et al., 2015; Lombard & Ditton, 1997; Riva et al., 2007). This could potentially counterbalance its relaxing effect. This hypothesis is supported by the greater cognitive activation observed in the two IVR conditions, as reported in previous studies (Jost et al., 2019; Wu et al., 2020). Furthermore, comments collected from our patients emphasized their tendency to move and explore more in the IVR conditions than in the narrative condition, indicating a greater commitment of attentional and visual resources. This can be particularly challenging for patients with PAH who often experience subthreshold cognitive issues, such as memory, attention, concentration, and executive function problems caused by chronic hypoxaemia and/or other factors, such as medication, comorbidities, and the overall impact of living with a chronic condition (Heller et al., 2023). Nevertheless, it is reasonable to conclude that the reduction in stress over time is evidence that the IVR experiences did not prevent relaxation. An alternative explanation is that the stress reduction was not a result of the interventions; it may instead have been caused by another factor, such as simply sitting in a chair for 10 minutes. Given the speculative nature of these considerations, further research is needed to explore the effectiveness of IVR in inducing relaxation in clinical populations, and in patients with PAH in particular. While for the IVR‐PS, we opted for the well‐tested ‘Secret Garden’ environment, which was specifically developed for relaxation experiences, the use of a preferred IVR to induce relaxation is a novel approach in this type of study and is supported by the idea that personalizing VR‐based scenarios is key to facilitating users' sense of presence and relaxation. We found no difference in perceived or physiological stress reduction. However, 88% of participants found the IVR‐PR extremely engaging, compared with 75% for the IVR‐PS. Similarly, 81% versus 75% rated the experience as being of an extremely high quality. Furthermore, 81% of participants found the preferred scenario to be more relaxing and enjoyable than the ‘Secret Garden’ scenario. This is consistent with a recent study by Pardini et al. (Pardini et al., 2022), which shows that users prefer personalized virtual environments. It is also important to note that, in the VR‐PR condition, patients were only offered the option of choosing a preferred scenario from a limited selection. Selecting an optimal preferred scenario, such as a VR environment that accurately replicates a location that induces relaxation for that specific user, could produce different and more significant results and should be investigated in future studies.

The lack of a significant difference in actual stress, combined with the results of the subjective user experience, suggests that combining relaxation induction— a method that has already been validated as an effective stress reducer—with immersive technology is more about making the technique more appealing, motivating or acceptable to users than about increasing its effectiveness.

Finally, we found no significant correlation between the degree of mental imagery and the impact of the varying relaxation techniques. However, none of the participants exhibited aphantasia and their scores were very similar. Therefore, more extensive research is required to investigate this aspect.

This pilot study has several limitations. These include a small, single‐centre, female‐only cohort; brief, single‐session interventions; a ‘preference’ option limited to two natural scenes (falling short of true personalization); a lack of blinded outcome assessment; possible expectancy effects; reliance on HR rather than comprehensive heart rate variability (HRV) indices; and uncertain ecological validity beyond inpatient rehabilitation. However, this study is the first to compare VR‐based relaxation with traditional narratives in patients with PAH in a rehabilitation setting, analysing both perceived and physiological effects, inducing stress before relaxation and comparing standard and preferred IVR scenarios. Moreover, as a feasibility study, it has provided several insights that will directly inform the design of a future randomized controlled trial (RCT). First, the trial confirmed that IVR relaxation is both feasible and well accepted among inpatients with PAH, indicating that recruitment and adherence within this population are achievable. Second, participants' higher engagement and willingness to reuse in the preference‐based IVR condition suggest that personalization should constitute a distinct intervention arm in the RCT. Third, the observed trends in HR and subjective distress reductions after IVR sessions support the use of post‐stress ΔHR and ΔSUDS as primary outcomes, with HRV indices and self‐reported relaxation as secondary endpoints. Finally, the variability detected in these measures offers empirical parameters for sample size estimation and stratification (e.g. by disease severity and imagery ability). Future studies should also adopt mixed‐methods designs that incorporate qualitative user experience (UX) interviews to explore reasons for scene selection (e.g. familiarity, autobiographical meaning, perceived restorative qualities or sensory preferences), test whether these mediate presence and outcomes, capture motivations, barriers and perceived mechanisms based on the effectiveness of specific conditions, examine durability via follow‐up and track adherence and reuse in home settings. Implementation‐oriented outcomes, including acceptability, feasibility, fidelity, and cost, will be essential for scaling up within PAH services.

Together, these findings provide a solid foundation for developing a larger RCT that will formally test the efficacy and mechanisms of personalized IVR interventions in PAH rehabilitation.

## CONCLUSIONS

Our results suggest that IVR and narrative are valuable methods for rehabilitating patients with PAH by reducing stress‐induced arousal. However, our findings also show that IVR does not significantly outperform traditional narrative techniques. Given IVR's higher costs, the lack of hospital technology, and the challenges of home use, these findings suggest that traditional methods should be relied upon for patients requiring relaxation during hospital stays, rehabilitation, or at home when VR facilities are unavailable. However, as over half of patients prefer virtual experiences, IVR training should be prioritized to boost patient engagement where possible.

## AUTHOR CONTRIBUTIONS

**Alessandra Gorini:** Conceptualization; methodology; validation; resources; writing – original draft; writing – review and editing; data curation; supervision; funding acquisition; project administration. **Beatrice De Maria:** Methodology; validation; formal analysis; data curation; writing – original draft. **Sara Checchia:** Investigation; writing – original draft. **Roberta Maioli:** Investigation. **Martina Vigorè:** Investigation. **Patrycja Krasinska:** Investigation; resources. **Maurizio Bussotti:** Resources; writing – review and editing. **Luca Bernardelli:** Software; resources. **Laura Adelaide Dalla Vecchia:** Writing – review and editing; supervision. **Stéphane Bouchard:** Conceptualization; methodology; writing – review and editing; supervision; funding acquisition.

## FUNDING INFORMATION

This research was supported by Premio Canada—Italia per l'Innovazione—Embassy of Canada and the Canada Research Chairs programme. The funding agencies had no role in the design of the study.

## Data Availability

The data that support the findings of this study are available from the corresponding author upon reasonable request.
